# Effect of Premorbid Antiplatelet Medication Use on Delayed Cerebral Ischemia After Aneurysmal Subarachnoid Hemorrhage: A Propensity Score-matched Study

**DOI:** 10.7759/cureus.5603

**Published:** 2019-09-09

**Authors:** Alejandro Enriquez-Marulanda, Mohamed M Salem, Krishnan Ravindran, Luis C Ascanio, Georgios A Maragkos, Santiago Gomez-Paz, Abdulrahman Y Alturki, Christopher S Ogilvy, Ajith J. Thomas, Justin Moore

**Affiliations:** 1 Neurology, Beth Israel Deaconess Medical Center / Harvard School of Medicine, Boston, USA; 2 Neurosurgery, Beth Israel Deaconess Medical Center/ Harvard Medical School, Boston, USA; 3 Neurosurgery, Beth Israel Deaconess Medical Center, Boston, USA; 4 Neurosurgery, Beth Israel Deaconess Medical Center / Harvard School of Medicine, Boston, USA; 5 Neurosurgery, Icahn School of Medicine at Mount Sinai, New York, USA

**Keywords:** antiplatelet, acetylsalicylic acid, aspirin, aneurysm, delayed cerebral infarction, infarction, subarachnoid hemorrhage, vasospasm

## Abstract

Introduction

Delayed cerebral ischemia (DCI) is a serious complication of aneurysmal subarachnoid hemorrhage (aSAH) and a major predictor of poor functional outcomes in patients surviving the initial insult. Several theories have postulated that platelet activation, microthrombi formation, and subsequent vasospasm are mechanisms involved. We, therefore, assessed the effect of premorbid antiplatelet medication (APM) use on discharge functional outcomes and cerebral infarction due to DCI in patients presenting with aSAH.

Methods

Retrospective analysis of patients admitted to a single US center with aSAH from 2007 to 2016 was performed. Patients who were receiving APM prior to admission were then matched to those who did not receive them using nearest-neighbor propensity-score-matching (PSM) controlling for the following variables: age, hypertension, smoking status, Hunt-&-Hess classification, and management type.

Results

Out of the 267 patients identified, 38 (14.2%) were on APMs when admitted. On univariate analysis, patients on APM were older (*p *< 0.001) and more likely to be hypertensive (*p *= 0.005). Modified Rankin Scale (mRS) at discharge was significantly worse for patients on APMs compared to those who were not (mRS 3-6 in 55.3% vs 32.7%; *p *= 0.007). No significant difference in cerebral infarction due to DCI was found (*p *= 0.82). PSM resulted in 20 patients in the APMs group and 20 patients in the comparison group. After matching, no significant difference was found in discharge mRS (*p *= 0.56) and cerebral infarction due to DCI (*p *= 0.7).

Conclusion

This study identified no significant effect of admission APMs on discharge functional outcomes and cerebral infarction due to DCI in aSAH patients after matching.

## Introduction

Aneurysmal subarachnoid hemorrhage (aSAH) is responsible for 2% to 5% of all new strokes and affects 21,000-33,000 people every year in the United States alone [1-2]. Aneurysm rupture triggers a local and systemic cascade of events that may lead to a pro-inflammatory, pro-coagulant, and pro-vasospastic state that may promote an ischemic environment [3]. One of the most feared complications of aSAH is delayed cerebral ischemia (DCI) with subsequent cerebral infarction, which is the most important predictor of poor functional outcomes in patients surviving the initial insult. DCI affects approximately 30% of aSAH patients and leaves the majority of survivors with significant cognitive deficits and reduced quality of life [4].

Several theories have been postulated to explain the nature of this complication including platelet activation, microthrombus formation, inflammatory response activation, and subsequent vasospasm [3,5-6]. While some attention has been drawn to the potential anti-inflammatory properties of antiplatelet agents and their effect on the pathophysiology of aneurysm growth and rupture, the role of antiplatelets in influencing the clinical course of aSAH patients remains to be explored [7-8]. While some data suggest that short-term use of antiplatelet agents may increase the risk of aSAH, it is unclear whether they may also provide a benefit through altering the pathophysiology of DCI [9]. We, therefore, sought to assess the effect of the use of premorbid antiplatelet medications (APMs) on functional outcomes at discharge and rates of DCI in patients presenting with aSAH in a single-center retrospective and propensity score-matched study.

## Materials and methods

Patient selection

**Figure 1:**
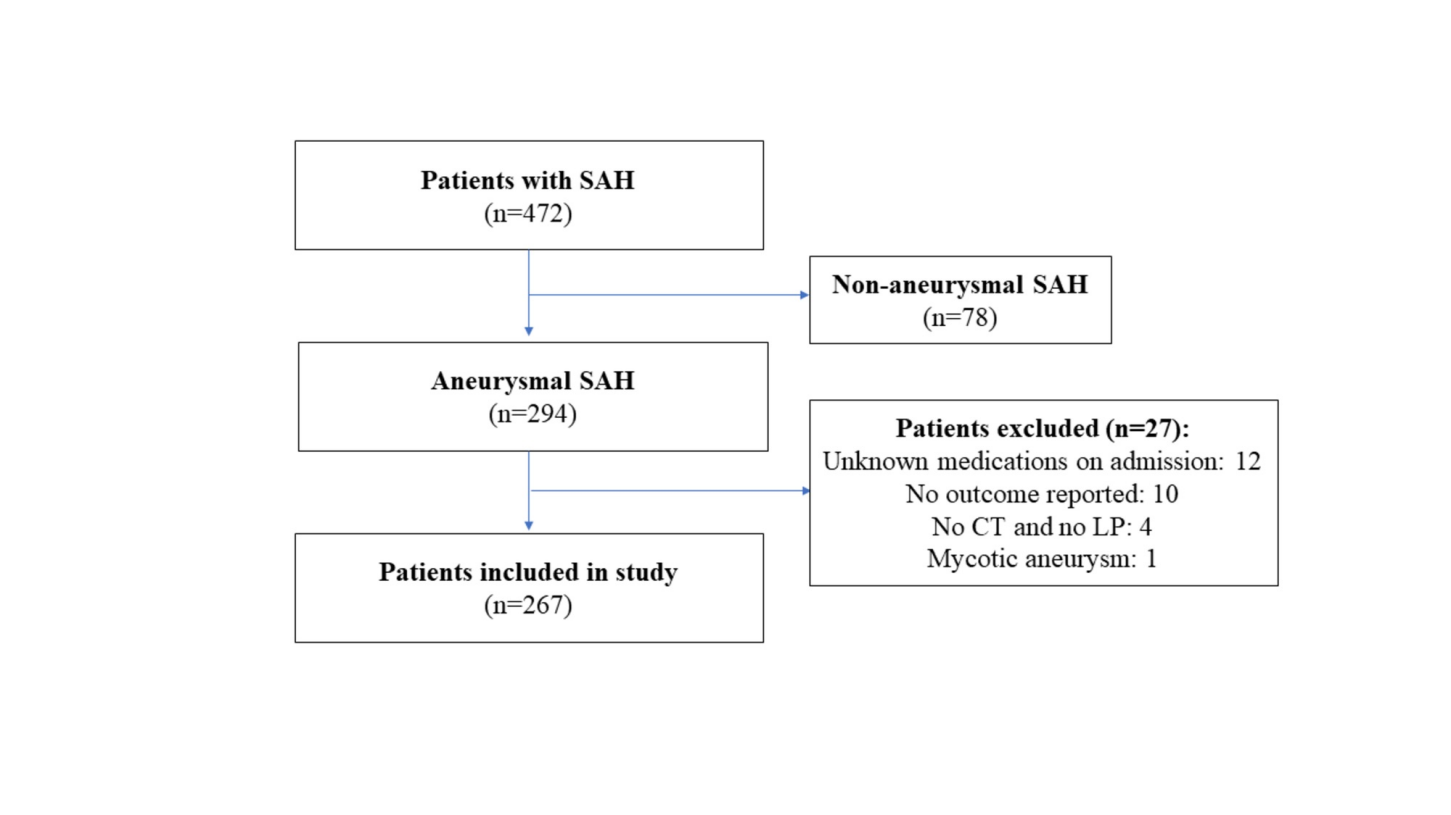
Flowchart of patients included in the present study SAH, subarachnoid hemorrhage; CT, computed tomography

Institutional review board approval was obtained prior to study initiation. We conducted a retrospective cohort comparison study consisting of all aSAH patients treated at a single major academic institution in the United States between 2007 and 2016. Patients diagnosed with aSAH on admission computed tomography (CT) scan or via detection of xanthochromia in cerebrospinal fluid (CSF) analysis were included. Exclusion criteria included incomplete documentation of admission medications and outcome on discharge, and patients with non-aneurysmal SAH or mycotic aneurysms were excluded (Figure 1).

Endpoints

The primary endpoint evaluated was the composite endpoint of death or disability on discharge, defined as scores of 3-6 on the modified Rankin scale (mRS). An mRS score of 0-2 corresponded to a good outcome with no or minimal neurologic symptoms, scores of 3-5 indicated poor outcome with increasing degrees of disability, while a score of six is death. The secondary endpoint evaluated was cerebral infarction due to DCI. This was defined as the presence of cerebral infarction on CT or magnetic resonance imaging (MRI) scan of the brain within 6 weeks of ictus, not present on initial CT or MR scan 48 hours after early aneurysm occlusion, and additionally not attributable to procedural-related infarctions (e.g. surgical clipping, endovascular treatment or external ventricular drain [EVD] placement} [4].

Data collection

The following patient parameters were recorded: demographic characteristics (age, gender, APM use at admission, anticoagulation use at admission, previous SAH), aneurysm characteristics (maximal diameter, anterior vs posterior circulation, side, multiple aneurysms), admission characteristics (systolic blood pressure, Glasgow Coma Scale [GCS], focal neurological deficit, World Federation of Neurological Societies [WFNS] score, Hunt and Hess [H&H] classification) and treatment during the hospitalization (placement of EVD device; placement of ventriculoperitoneal [VP] shunt), and aneurysm management (medical therapy, microsurgical clipping, endovascular therapy, both microsurgical clipping and endovascular therapy; Table 1).

Radiologic characteristics on admission CT (modified Fisher score; SAH thickness defined according to the BNI scale as perpendicular to the direction of the cistern or fissure on axial cut in the thickest-appearing region; diffuse cerebral edema, defined as collapsed sulci in ≥50% of one hemisphere, or collapse of the basal cisterns; presence of intraparenchymal hemorrhage (IPH); IPH location; IPH volume (calculated as A*B*C/2 where A is the greatest hemorrhage diameter, B is the diameter 90° to A, and C is the rostrocaudal maximal diameter); and presence of hydrocephalus [10]. Clinical outcome as mRS was acquired from discharge notes. Admission GCS, WFNS, and H&H scores were reported by the neurosurgeon on first contact with the patient. IPH volume, SAH thickness, cerebral edema, hydrocephalus, and cerebral infarction were retrospectively measured on the CT/MRI scans.

Statistical analyses

Categorical variables were reported as proportions. Continuous variables were reported as mean ± standard deviation (SD) or median (Interquartile range [IQR]) as appropriate according to the distribution of the data. In each group, categorical variables were compared using the chi-square test, and continuous variables were compared using the Mann-Whitney U test. Observational treatment studies are limited by lack of randomization, and factors influencing treatment selection may also influence outcome, leading to bias. Therefore, we performed an analysis with propensity score matching (PSM) using the nearest-neighbor method with replacement controlling for age, hypertension, smoking status, H&H classification, and aneurysm management type. PSM uses a linear combination of covariates for calculating a single score and then ensures that the treatment groups are balanced with respect to the measured covariates. Statistical significance was set at *p*-value <0.05. All statistical analyses were performed using the Stata 14 statistical software package (StataCorp., College Station, TX).

## Results

Patient characteristics

A total of 267 patients were included in this analysis. Baseline characteristics are presented in Table 1. The mean age was 57.4 ± 14.6 years. The majority of patients were female (66.3%) and active smokers (40.8%). Thirty-eight (14.2%) patients were receiving APMs therapy on admission, primarily aspirin (ASA; n = 36) with 2 on a dual antiplatelet regimen (including ASA). From the APM group, only 12 (31.6%) received a platelet apheresis transfusion. The APMs were continued after securing the aneurysm in 16 (42.1%) patients, all of them had an endovascular approach. Anticoagulation was present in 4 (1.5%) cases, only one of these having both anticoagulation and antiplatelet medications at admission. The median modified Fisher CT scale grade was 4 (IQR: 3-4), with 183 (68.6%) patients presenting with intraventricular hemorrhage (grade 2 or 4). Median SAH thickness was 6.9 mm (IQR: 5.08 to 9.42). A BNI score of 3 was most commonly reported (153 cases; 57.3%). An EVD was placed in 147 (55.1%) cases. Cerebral infarction due to DCI was identified in 39 (14.6%) of cases. Discharge mRS was 0 for 129 patients (48.3%), 1 for 19 (7.1%), 2 for 23 (8.6%), 3 for 26 (9.7%), 4 for 16 (6.0%), 5 for 7 (2.6%), and 6 (death) for 47 patients (17.6%).

**Table 1:** Baseline sociodemographic and clinical characteristics Missing data (values that were not stored for a variable in the observation of interest): (a) 54 (b) 9 (c) 40 (d) 1 ASA: acetylsalicylic acid; LMWH: low molecular weight heparin; SBP: systolic blood pressure; WFNS: World Federation of Neurosurgical Societies; GCS: Glasgow coma scale; SAH: subarachnoid hemorrhage; BNI: Barrow Neurological Institute; IPH: intraparenchymal hemorrhage; CHF: cardiac heart failure; EVD: external ventricular drain; VP: ventriculoperitoneal; DCI: delayed cerebral ischemia; mRS: modified Rankin scale

Variables	Result (n=267)
Age	57.4 (±14.6)
Gender	
Female	177 (66.3%)
Male	90 (33.7%)
Smoking (a)	87 (40.8%)
Chronic hypertension (b)	129 (50.0%)
Antiplatelet at presentation	38 (14.2%)
ASA	36 (94.7%)
ASA+Dypiridamole	1 (2.6%)
ASA+Clopidogrel	1 (2.6%)
Antiplatelet continued during hospitalization	n=38
Yes	16 (42.1%)
No	22 (57.9%)
Anticoagulation at presentation	4 (1.5%)
Warfarin	2 (50.0%)
LMWH	2 (50.0%)
SBP on admission (c)	137.7 (±25.7)
SBP on admission ≥140 (c)	107 (47.1%)
WFNS (d)	
1	120 (45.1%)
2	25 (9.4%)
3	4 (1.5%)
4	39 (14.7%)
5	78 (29.3%)
Hunt & Hess	
1	55 (20.6%)
2	69 (25.8%)
3	51 (19.1%)
4	49 (18.3%)
5	43 (16.1%)
Hunt & Hess (4-5)	92 (34.5%)
Modified Fisher	
1	12 (4.5%)
2	13 (4.9%)
3	72 (27.0%)
4	170 (63.7%)
GCS	14 (IQR 6 - 15)
Severe	104 (39.1%)
Moderate	13 (4.9%)
Mild	149 (56.0%)
SAH thickness (mm)	6.9 (IQR 5.04 to 9.3)
BNI score	2.9 (±0.72)
1	2 (0.7%)
2	64 (23.9%)
3	153 (57.3%)
4	41 (15.4%)
5	7 (2.6%)
Cerebral edema	34 (12.7%)
IPH presence	56 (21.0%)
Volume IPH (mL3)	8.22 (IQR 2.62 to 18.79)
IPH location	n=56
Lobar cerebral	55 (98.2%)
Cerebellum	1 (1.8%)
Seizure during hospitalization	22 (9.0%)
Cardiac abnormality in first 24 hours	12 (4.5%)
Atrial fibrillation	3 (25.0%)
Acute CHF	1 (8.3%)
Acute Myocardial Infarction	4 (33.3%)
Pulseless electrical activity	1 (8.3%)
Takotsubo cardiomyopathy	1 (8.3%)
Ventricular Fibrillation/Tachycardia	2 (16.7%)
Hydrocephalus on admission (d)	125 (47.0%)
EVD in first 24 hours	147 (55.1%)
VP shunt placement	45 (16.8%)
DCI-induced Cerebral infarction	39 (14.6%)
mRS at discharge	
0	129 (48.3%)
1	19 (7.1%)
2	23 (8.6%)
3	26 (9.7%)
4	16 (6.0%)
5	7 (2.6%)
6	47 (17.6%)

Aneurysm characteristics and treatment

The majority of aneurysms (92.9%) had saccular morphology. The median aneurysm maximum diameter was 5 mm (IQR: 3.8 to 7.3). Medical management alone was instituted in 26 (9.7%) cases, while the rest of patients underwent either endovascular embolization (n = 195; 73%), microsurgical clipping (n = 42; 15.7%), or both clipping and endovascular embolization (n = 4; 1.5%). Aneurysm characteristics are presented in Table 2.

**Table 2:** Intracranial aneurysms characteristics Max: maximum; PED: pipeline embolization device

Variables	Result (n=267)
Aneurysm morphology	
Saccular	248 (92.9%)
Fusiform/Blister	19 (7.1%)
Circulation location	
Anterior	209 (78.3%)
Posterior	58 (21.7%)
Aneurysm Max. Size (mm)	5 (IQR 3.8 to 7.3)
Multiple aneurysms	3 (1.1%)
Aneurysm management	
Medical	26 (9.7%)
Microsurgical clipping	42 (15.7%)
Endovascular therapy	195 (73.0%)
Clipping+Endovascular	4 (1.5%)
Endovascular treatment type	n=199
Coiling	189 (95.0%)
Stent-Coil	6 (3.0%)
PED	3 (1.5%)
Onyx	1 (0.5%)

Unmatched comparison according to APMs therapy

In unmatched univariate analysis (Table 3), aSAH patients that were on APMs on presentation were older (68.5 vs 55.5 years old; *p *< 0.001) and more likely to have hypertension (71% vs 46.4%; *p *= 0.005). Poor neurological outcomes on discharge were significantly higher among patients with APMs (55.3% vs 32.7%; *p *= 0.007). SAH thickness was similar between the two cohorts (7.5 mm in the APM cohort vs 6.8 mm in the non-APM; *p *= 0.08). Additionally, neither the presence of IPH nor its volume were significantly different between the two cohorts (*p *= 0.66 and *p *= 0.68, respectively). The rates of DCI-related cerebral infarctions were non-significantly different between the two groups (15.8% vs 14.4%; *p *= 0.82).

**Table 3:** Comparative analysis according to antiplatelet medication on admission before propensity-score matching p-value <0.05; n (%), p-value: Chi^2^ test; mean (±SD), p-value: two-sample T-test; median (IQR), p-value: Mann-Whitney U test SBP: Systolic blood pressure; WFNS: World Federation of Neurosurgical Societies; GCS: Glasgow coma scale; SAH: subarachnoid hemorrhage; IPH: intraparenchymal hemorrhage; CHF: cardiac heart failure; EVD: external ventricular drain; VP: ventriculoperitoneal; DCI: delayed cerebral ischemia; mRS: modified Rankin scale

Variables	Antiplatelets		p-Value
	No 229 (85.8%)	Yes 38 (14.2%)	
Age	55.5 (±13.9)	68.5 (±13.8)	<0.001
Gender			
Female	151 (65.9%)	26 (68.4%)	0.76
Male	78 (34.1%)	12 (31.6%)	
Smoking	77 (42.3%)	10 (32.3%)	0.29
Chronic hypertension	102 (46.4%)	27 (71.0%)	0.005
Anticoagulation on presentation	3 (1.3%)	1 (2.6%)	0.53
Aneurysm morphology			
Saccular	213 (93.0%)	35 (92.1%)	0.84
Fusiform	16 (7.0%)	3 (7.9%)	
Circulation location			
Anterior	178 (77.7%)	31 (81.6%)	0.59
Posterior	51 (22.3%)	7 (18.4%)	
Aneurysm Max. Size (mm)	5 (IQR 3 .8 - 7)	5 (IQR 4 - 8)	0.89
Multiple aneurysms	3 (1.3%)	0 (0.0%)	0.48
SBP on admission	137.8 (±26.4)	137.1 (±21.6)	0.89
SBP on admission ≥140	90 (46.1%)	17 (53.1%)	0.46
WFNS			
1	106 (46.5%)	14 (36.8%)	0.25
2	21 (9.2%)	4 (10.5%)	
3	2 (0.9%)	2 (5.3%)	
4	32 (14.0%)	7 (18.4%)	
5	67 (29.4%)	11 (28.9%)	
Hunt & Hess			
1	46 (20.1%)	9 (23.7%)	0.85
2	62 (27.1%)	7 (18.4%)	
3	43 (18.8%)	8 (21.0%)	
4	42 (18.3%)	7 (18.4%)	
5	36 (15.7%)	7 (18.4%)	
Hunt & Hess (4-5)	78 (34.1%)	14 (36.8%)	0.74
Modified Fisher			
1	11 (4.8%)	1 (2.6%)	0.44
2	13 (5.7%)	0 (0%)	
3	61 (26.6%)	11 (28.9%)	
4	144 (62.9%)	26 (68.4%)	
GCS	14 (IQR 6 - 15)	13.5 (IQR 6 - 15)	0.61
Severe	87 (38.2%)	17 (44.7%)	0.63
Moderate	12 (5.3%)	1 (2.6%)	
Mild	129 (56.6%)	20 (52.6%)	
SAH thickness (mm)	6.8 (IQR 4.96 - 9.13)	7.52 (IQR 5.98 - 10.1)	0.08
Cerebral edema	28 (12.3%)	6 (15.0%)	0.63
IPH presence	47 (20.5%)	9 (23.7%)	0.66
Volume IPH (mL3)	8.14 (IQR 2.66 - 18.06)	8.57 (IQR 2.58 - 19.56)	0.68
Seizure during hospitalization	19 (9.05%)	3 (8.8%)	0.97
Cardiac abnormality in first 24 hours	9 (3.9%)	3 (7.9%)	0.27
Hydrocephalus on admission	108 (47.4%)	17 (44.7%)	0.76
EVD in first 24 hours	122 (53.3%)	25 (65.8%)	0.15
VP shunt placement	38 (16.6%)	7 (18.4%)	0.78
Aneurysm management			
Medical	21 (9.2%)	5 (13.2%)	0.17
Microsurgical clipping	36 (15.7%)	6 (15.8%)	
Endovascular	170 (74.2%)	25 (65.8%)	
Clipping+Endovascular	2 (0.9%)	2 (5.3%)	
Cerebral infarction due to DCI	33 (14.4%)	6 (15.8%)	0.82
mRS at discharge			
0 to 2	154 (67.3%)	17 (44.7%)	0.007
3 to 6	75 (32.7%)	21 (55.3%)	

Comparison according to APMs therapy after propensity-score matching

Propensity score matching was performed controlling for age, hypertension, smoking status, H&H classification, and management type. Matching identified 20 patients from each cohort. The rate of cerebral infarction due to DCI was slightly lower in patients with APMs therapy, without achieving statistical significance (20% vs 25%; p = 0.7). Poor neurological outcomes on discharge were slightly higher among the patients with APMs therapy, again not reaching statistical significance (40% vs 30%; p = 0.7). No significant differences were noted on other patient, aneurysm, or treatment characteristics (Table 4).

**Table 4:** Comparative analysis according to antiplatelet medication on admission after propensity score matching adjusted for age, hypertension, smoking status, Hunt & Hess classification, and management type p-value <0.05; n (%), p-value: Chi^2^ test; mean (±SD), p-value: two-sample T-test; median (IQR), p-value: Mann-Whitney U test SBP: systolic blood pressure; WFNS: World Federation of Neurosurgical Societies; GCS: Glasgow coma scale; SAH: subarachnoid hemorrhage; IPH: intraparenchymal hemorrhage; CHF: cardiac heart failure; EVD: external ventricular drain; VP: ventriculoperitoneal; DCI: delayed cerebral ischemia; mRS: modified Rankin scale

Variables	Antiplatelets		p-Value
	No 20 (50.0%)	Yes 20 (50.0%)	
Age	62.1 (±13.8)	62.7 (±12.0)	0.87
Gender			
Female	15 (75.0%)	13 (65.0%)	0.49
Male	5 (25.0%)	7 (35.0%)	
Smoking	8 (40.0%)	6 (30.0%)	0.51
Chronic hypertension	14 (70.0%)	13 (65.0%)	0.74
Anticoagulation on presentation	0 (0.0%)	1 (5.0%)	0.31
Aneurysm morphology			
Saccular	17 (85.0%)	18 (90.0%)	0.63
Fusiform	3 (15.0%)	2 (10.0%)	
Circulation location			
Anterior	15 (75.0%)	15 (75.0%)	>0.99
Posterior	5 (25.0%)	5 (25.0%)	
Aneurysm Max. Size (mm)	5 (IQR 3.4 - 7)	5 (IQR 4 - 7.2)	0.87
Multiple aneurysm	2 (10.0%)	0 (0%)	0.15
SBP on admission	136.2 (±28.3)	137.7 (±19.7)	0.84
SBP on admission ≥140	10 (55.6%)	10 (50.0%)	0.73
WFNS			
1	11 (55.0%)	8 (40.0%)	0.38
2	2 (10.0%)	1 (5.0%)	
3	0 (0%)	2 (10.0%)	
4	2 (10.0%)	5 (25.0%)	
5	5 (25.0%)	4 (20.0%)	
Hunt & Hess			
1	1 (5.0%)	5 (25.0%)	0.31
2	8 (40.0%)	4 (20.0%)	
3	5 (25.0%)	3 (15.0%)	
4	4 (20.0%)	5 (25.0%)	
5	2 (10.0%)	3 (15.0%)	
Hunt & Hess (4-5)	6 (30.0%)	8 (40.0%)	0.51
Modified Fisher			
1	0 (0.0%)	1 (5.0%)	0.57
2	1 (5.0%)	0 (0%)	
3	4 (20.0%)	4 (20.0%)	
4	15 (75.0%)	15 (75.0%)	
GCS	15 (IQR 6.5 - 15)	14 (IQR 7 - 15)	0.62
Severe	7 (35.0%)	9 (45.0%)	0.52
Moderate	0 (0.0%)	0 (0.0%)	
Mild	13 (65.0%)	11 (55.0%)	
SAH thickness (mm)	6.6 (IQR 4.8 - 8.9)	7.5 (IQR 4.9 - 9.1)	0.57
Cerebral edema	2 (10.0%)	4 (20.0%)	0.38
IPH presence	5 (25.0%)	6 (30.0%)	0.72
Volume IPH (mL^3^)	4.9 (IQR 2.2 - 12.1)	18.6 (IQR 8.6 - 51.8)	0.2
Seizure during hospitalization	2 (11.1%)	2 (10.5%)	0.95
Cardiac abnormality in first 24 hours	1 (5.0%)	1 (5.0%)	>0.99
Hydrocephalus on admission	9 (45.0%)	8 (40.0%)	0.75
EVD in first 24 hours	9 (45.0%)	12 (60.0%)	0.34
VP shunt placement	2 (10.0%)	2 (10.0%)	>0.99
Aneurysm management			
Medical	2 (10.0%)	2 (10.0%)	0.16
Microsurgical clipping	1 (5.0%)	6 (30.0%)	
Endovascular	16 (80.0%)	12 (60.0%)	
Clipping+Endovascular	1 (5.0%)	0 (0.0%)	
DCI-induced cerebral infarction	5 (25.0%)	4 (20.0%)	0.7
mRS at discharge			
0 to 2	14 (70.0%)	12 (60.0%)	0.51
3 to 6	6 (30.0%)	8 (40.0%)	

## Discussion

Given the prevalence of aneurysms in the general population and the devasting outcomes of rupture, the optimization of aneurysm management algorithms has attracted significant attention, particularly in the prevention of DCI-induced cerebral infarction. The cerebral microcirculation role in SAH is now increasingly recognized as a significant contributor in the pathophysiology of DCI [11-12]. Moreover, it may represent the key to understand cases with discrepancies between “angiographic” and “clinical” vasospasm. Notably, nimodipine, only drug currently approved for SAH, has not been shown to have an effect on angiographic vasospasm (which evaluates large vessels), despite being associated with reduced risk of poor outcome [3]. Platelet activation may have a crucial role in the derangement of the brain microcirculatory state generating a pro-ischemic state in aSAH after post-bleed day 3 [13].

Antiplatelet medications, particularly ASA, have the potential to interact with aneurysms at multiple levels, including aneurysm growth, rupture rates, and post-rupture outcomes through a variety of molecular pathways. This is particularly relevant, as population aging has led to the increasing use of APMs in the context of cardiovascular disease and ischemic stroke prevention. Importantly, stopping ASA does not result in immediate restoration of platelet activation and adhesion function to normal, as the mechanism of action involves the irreversible inhibition of cyclooxygenase enzymes, decreasing the release of thromboxane A2, with lasting effects up to 10 days [14]. Moreover, it has been reported that ASA inhibits vasoconstriction mediated by oxyhemoglobin [15]. Therefore, we sought to analyze the effects of pre-admission use of APMs on clinical outcomes after aneurysm rupture, initial hemorrhage thickness, and DCI-induced cerebral infarction rates. Currently, few literature reports have evaluated premorbid use of APMs, particularly ASA, and its effect on SAH (Table 5) [14,16-20].

Poor neurologic outcome

In our study, we found a small but non-significant increase in the rates of poor neurologic outcomes in the APMs group. Toussaint et. al first reported that ASA use had no significant effect on overall outcome following SAH, suggesting that the presence of aneurysm was not a contraindication to ASA therapy [16]. Likewise, a study performed by Bruder et al. that also performed a matched-pair analysis of 1422 aSAH patients found small (non-significant) increased rates of poor neurologic outcomes in the ASA cohort [18].

A multicenter Japanese registration study reported an age-dependent differential response in SAH patients to pre-hemorrhage antiplatelet use, with improved outcomes in patients <60 years, and significantly worsened outcomes in patients >60 years old [21]. However, “poor outcome” was defined by the authors as an mRS ≥4, in contrast to other studies including ours, which define it as an mRS >2, which limits comparisons. Additionally, they did not report overall rates of poor neurologic outcome based on APMs use and only reported p-values from age-stratified comparisons.

ASA responder status may contribute to these non-conclusive results. A study performed by Von der Brelie et al. that compared the outcome results according to impairment of platelet function testing in patients with ASA use pre-rupture showed that patients with impaired platelet function testing in the context of ASA use had non-significantly higher rates of poor neurologic outcomes [20] (**Table 5**).

**Table 5:** Literature review of premorbid antiplatelet medications use in aneurysmal subarachnoid hemorrhage and its effect on delayed cerebral ischemia-induced cerebral infarction and poor neurologic outcome (a) Used as an outcome measure the modified Glasgow Outcome Scale (Poor neurologic outcome was defined as GOS≤3) instead of validated modified Rankin scale (b) Groups were categorized based on resistance to aggregation on platelet function testing (considered as a better marker of APM use rather than the history of medication administration) (c) Used as an outcome measure the NIS-SOM (Composite NIS-SAH outcome measure) instead of validated modified Rankin scale N/A: Outcome not reported in the study and unable to calculate it with the data available; APMs: antiplatelet medications; DCI: delayed cerebral ischemia; GOS: Glasgow outcome scale [14,16-20]

Author (Year)	Type of study	Sample size	premorbid APMs users	APMs non-users	DCI-induced cerebral infarction		p-Value	Poor neurologic outcome		p-Value
					APMs use	No APMs		APMs use	No APMs	
Juvela (1995)	Prospective study	195	85	110	2.4%	12.7%	0.009	N/A	N/A	N/A
Toussaint (2004)	Retrospective study (a)	305	29	276	N/A	N/A	N/A	34.0%	33.0%	0.87
Gross (2014)	Retrospective study	274	32	242	13.0%	14.0%	>0.99	N/A	N/A	N/A
Von der Brelie (2017)	Retrospective study (a,b)	79	55	24	34.5%	62.5%	0.02	69.10%	66.70%	0.83
Dasenbrock (2017)	Big data study (c)	11,549	108	11,441	N/A	N/A	N/A	36.10%	37.80%	0.07
Bruder (2018)	Retrospective study, matched analysis	1,422	144	1278 (144 after matching)	44.4%	41.7%	0.7	50.70%	47.90%	0.7
Enriquez-Marulanda (2018)	Retrospective study, matched analysis	267	38 (20 after matching)	229 (20 after matching)	20.0%	25.0%	0.7	40.0%	30.0%	0.51

Only the study performed by Dasenbrock et al., a Nationwide Inpatient Sample database study of 11,549 SAH patients, identified an overall non-significant lower rate of poor neurologic outcome in the APMs group [19]. Long-term ASA users had lower odds of longer hospital stay and cardiac complications. However, subgroup analysis revealed that these benefits were mainly restricted to patients treated endovascularly [19]. Notably, only patients who underwent intervention were included in this study, excluding other SAH subgroups (e.g. initial poor grade on admission, etc.). This study is considered as part of the recent tide of “big data” papers, which have the advantage of conducting research using large ready-made administrative databases that include data from many institutions and is able to detect even small differences. A drawback, of these analyses, however, is that these databases were not designed for research purposes and the variables were not defined by neurosurgeons for the study of neurosurgical diseases [22]. Indeed, these datasets are inputted by administrative personnel, in nonclinical settings, and without medical knowledge in contrast to neurosurgical department databases [22].

Severity Of Initial Hemorrhage

Inhibition of platelet function may be counterproductive in SAH as there is a theoretical risk of hemorrhage exacerbation through inhibiting platelet aggregation, additionally, this may increase the risk of rebleeding and increase the risk of other bleeding complications after surgical procedures. The reported risk of ASA-associated intracerebral hemorrhage, however, has varied from one study to another but is consistently low [23]. In our cohort, ASA users had a non-significant trend towards greater SAH clot thickness and IPH volumes, a phenomenon that requires exploration in larger studies. In contrast to our study, previous literature on premorbid APMs use in aSAH has not studied the effect on the risk of exacerbation of initial subarachnoid clot thickness.

Cerebral Infarction Due To Delayed Cerebral Ischemia

DCI and subsequent infarction are increasingly recognized as key predictors of outcome in patients surviving the acute stage of SAH [3,5]. While putatively linked to vasospasm, mounting evidence suggests that DCI is, in reality, a multifactorial process. Initial brain insult induces arteriolar vasospasm with subsequent formation of microthrombi - as a result of platelet activation and aggregation and associated endothelial injury that promotes the secretion of granules with inflammatory, thrombogenic and vasoactive mediators [4,6,13,24-26]. This leads to a perfusion mismatch and neurovascular uncoupling resulting in astrocyte apoptosis and further endothelial dysfunction. Concurrently, lysis of subarachnoid clot results in oxidative stress and induces arteriolar smooth muscle necrosis, promoting migration of inflammatory cells, eventually resulting in infarction.

The pivotal role played by inflammatory signaling and activation of platelet pathways suggests antiplatelet agents may be useful as potential therapeutic targets in DCI. The first study that evaluated the pre-rupture use of an antiplatelet agent in SAH patients found a significantly lower rate of DCI-induced cerebral infarction in the ASA group (Table 5) [14]. Comparison based on ASA responder status also found a significantly lower rate of DCI-induced cerebral infarction in the group on premorbid APMs and had an impaired platelet function testing on admission [20]. Despite the significant benefit in terms of DCI-induced cerebral infarction rates of APMs use shown in the previous studies and the evidence implicating microthrombi in the underlying pathophysiology behind DCI, our propensity score-matched study, failed to show significant benefit [27]. Additionally, two retrospective studies found no significant benefit of APMs in aSAH for the prevention of DCI-induced cerebral infarction [17-18]. In the study by Dasenbrock et al., unfortunately, the incidence of DCI and cerebral infarction were not assessed by the authors [19].

Controversy of the benefit of APMs in SAH also has been present when given as treatment after securing the intracranial aneurysm due to conflicting results of prospective clinical studies. While a meta-analysis of all randomized trials of platelet aggregation inhibitors in SAH suggested a decrease in DCI with a trend of better outcomes in patients treated with ASA [28], a follow-up phase III randomized trial failed to confirm the benefit [29]. Recently, Nagahama and colleagues have shown that patients with subarachnoid hemorrhage treated endovascularly and requiring dual antiplatelet therapy had a significantly lower risk of vasospasm and DCI [30].

The additive effect of irreversible inhibition of the P2Y12 ADP receptor by clopidogrel and ASA in inhibiting platelet aggregation is well-established, and thus it is likely that dual antiplatelet therapy markedly reduces microthrombi formation. However, in our series, the majority of patients were treated with coil-embolization, and thus did not receive dual antiplatelet therapy. Only two patients received dual therapy, thus limiting any significant subgroup analysis. In the future, larger and prospective studies would be required to determine the effect of dual antiplatelet agents on the outcome of patients with aSAH.

Limitations

This study is limited by its retrospective non-randomized nature with all the inherent bias of such a study design. APM exposure duration prior to the aSAH was not available in the electronic records preventing detailed analysis. Although a large number of aSAH occurred during the study period, the sample size of this study was relatively small after matching, which may limit the ability to detect differences between groups. While PSM is purported to have a similar effect to randomization, this technique only ensures balance in observed covariates, whereas randomization balances both observed and unobserved covariates.

## Conclusions

This propensity-matched study showed no significant effect of premorbid APMs on discharge functional outcomes and cerebral infarction due to DCI in presenting with aSAH. ASA users had greater SAH clot thickness and IPH volumes; however, this trend was not statistically significant. 
